# Exposure to different residential indoor characteristics during childhood and asthma in adolescence: a latent class analysis of the Danish National Birth Cohort

**DOI:** 10.1007/s10654-023-01051-y

**Published:** 2023-10-21

**Authors:** Amélie Keller, Jonathan Groot, Clara Clippet-Jensen, Angela Pinot de Moira, Marie Pedersen, Torben Sigsgaard, Steffen Loft, Esben Budtz-Jørgensen, Anne-Marie Nybo Andersen

**Affiliations:** 1 Section of Epidemiology, Department of Public Health, University of Copenhagen, Copenhagen, Denmark; 2 Environment, Work and Health, Department of Public Health, University of Aarhus, Aarhus, Denmark; 3 Section of Environmental Health, Department of Public Health, University of Copenhagen, Copenhagen, Denmark; 4 Section of Biostatistics, Department of Public Health, University of Copenhagen, Copenhagen, Denmark; 5 National Heart and Lung Institute, Imperial College, London, United Kingdom

**Keywords:** Home characteristics, Indoor air pollution, Danish National Birth Cohort, Environmental epidemiology, Asthma

## Abstract

**Background:**

Many residential indoor environments may have an impact on children’s respiratory health.

**Objectives:**

The aims of this study were to identify latent classes of children from the Danish National Birth Cohort (DNBC) who share similar patterns of exposure to indoor home characteristics, and to examine the association between membership in the latent classes and asthma in adolescence.

**Methods:**

We included data on residential indoor characteristics of offspring from the DNBC whose mothers had responded to the child’s 11-year follow-up and who had data on asthma from the 18-year follow-up. Number of classes and associations were estimated using latent class analysis. To account for sample selection, we applied inverse probability weighting.

**Results:**

Our final model included five latent classes. The probability of current asthma at 18 years was highest among individuals in class one with higher clustering on household dampness (9, 95%CI 0.06–0.13). Individuals in class four (with higher clustering on pets ownership and living in a farm) had a lower risk of current asthma at age 18 compared to individuals in class one (with higher clustering on household dampness) (OR 0.53 (95%CI 0.32–0.88), *p* = .01).

**Conclusion:**

Our findings suggest that, in a high-income country such as Denmark, groups of adolescents growing up in homes with mold and moisture during mid-childhood might be at increased risk of current asthma at age 18. Adolescents who grew-up in a farmhouse and who were exposed to pets seem less likely to suffer from asthma by age 18.

**Supplementary Information:**

The online version contains supplementary material available at 10.1007/s10654-023-01051-y.

## Introduction

Asthma is a chronic non-communicable disease characterized by chronic airway inflammation and reversible airflow limitation [1]. Asthma is the most common pediatric chronic disease and its average prevalence varies greatly across regions: from 5.6 and 6.7% in South-East Asia and Western Pacific to over 13.6 and 21.7% in Spain among children aged 6–7 years and 13–14 years, respectively [2]. In the European Union (EU), its estimated prevalence is 9.4% among children [3]. Although asthma can develop at any age, the first symptoms most often appear during childhood [1]. The etiology of asthma development is complex and many risk factors, such as genetic, behavioral and environmental factors, have been associated with asthma development [4]. Among the environmental factors, increasing scrutiny has been directed at the potential adverse health effects of indoor air pollution, as European adults and children spend most of their time indoors [5]. Therefore, exposure to detrimental indoor environments may greatly impact individuals’ health and especially children’s. Indeed, although their vulnerability differs across age, children are more susceptible to harmful effects of air pollution than adults [6], partly due to their respiratory and immune systems as well as metabolic pathways being immature. In addition, children have higher breathing rates per unit of body weight than adults do, hence increasing their exposure [7].

Many residential indoor environments may have an impact on inhabitants’ health. In European homes, particulate matter (PM) nitrogen dioxide (NO_2_) can come from a variety of common indoor sources, such as tobacco smoking, wood-burning/gas stoves, candle-burning, fireplaces, cooking practices, appliance use and incenses. Microbial pollution due to excess moisture, water damage and growth of mold is associated with increased prevalence of respiratory symptoms, allergies and asthma [8–10]. In addition, location of the home in relation to outdoor sources of air pollution such as road traffic, wind exposure, the characteristics and age of the building, type of floor, ventilation system, dwelling size, activities in the home, heating and construction materials, furnishing, cleaning and household composition (i.e. density of individuals, animals) also contribute to microbial and PM pollution [11]. Findings from a systematic review from 2015 on the exposure to indoor pollutants, such as indicators of damp-induced bacteria or indoor mold exposure (i.e. endotoxins, Penicillium and Aspergillus (EPS-Pen/Asp)) or chemicals (NO_2_, volatile organic compounds (VOC), PM) and wheeze and asthma suggest that there is sufficient evidence for the positive association between endotoxin exposure and asthma and wheeze. On the other hand, the evidence of exposure to pet allergens and adverse respiratory health effects in early ages is limited and contradictive. Evidence of associations between VOCs, NO_2_, PM and formaldehyde and asthma and wheeze is classified as insufficient as studies suffer from multiple limitations such as lack of rigorous protocols and definition of outcomes or inappropriate population selection [12].

Because individuals are likely to be simultaneously exposed to several indoor pollutants sources and housing characteristics, associations with the risk of asthma can be difficult to disentangle and a multiple residential indoor environments approach might be helpful [13]. Latent class analysis (LCA) identifies groups of individuals with shared exposure profiles [14], which enables the identification of exposure combinations within individuals manifesting similar traits. In addition, in combination with other statistical models (i.e. logistic regression), estimation of elevated risk among one or more of these groups of individuals is possible. These properties are especially relevant for public health as it may facilitate the development of targeted interventions [15] among specific groups of individuals with shared characteristics.

The application of LCA in environmental epidemiology is not widespread and its use in modelling indoor pollutant sources and asthma is scarce. The aims of this study were, thus, to identify latent classes (i.e., clusters) of children from the Danish National Birth Cohort (DNBC) who share similar patterns of exposure to indoor home characteristics, and to examine the prospective association between membership in the latent classes and asthma in adolescence.

## Methods

This study followed the reporting principles of the Strengthening the Reporting of Observational Studies in Epidemiology (STROBE) statement [16].

### Data sources

#### Population

**Fig. 1:**
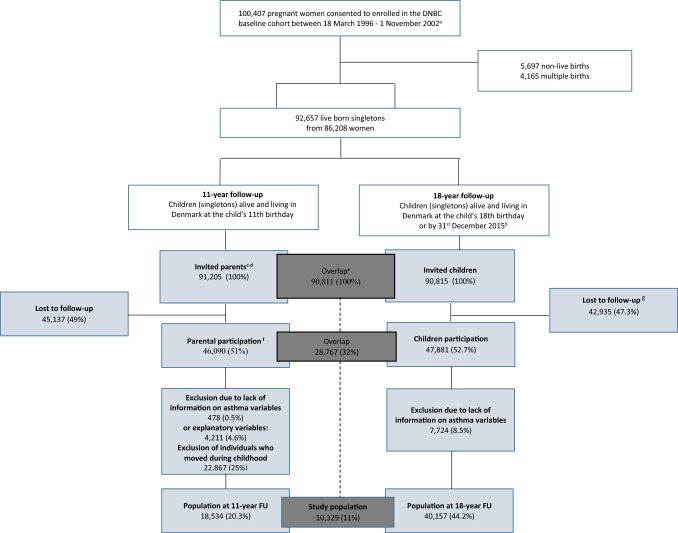
Flowchart of the invited and participating populations in the 11-year & 18-year data collections of the Danish National Birth Cohort **a** Individuals who have not withdrawn their consent as of December 2021. **b** The latest available update from registers was in 2015 when most DNBC children had not yet turned 18 years old **c** The invitation was mailed by postal service to the parent(s) for whom the child had residential address **d** One parent could answer more than one questionnaire if more than one of their children was enrolled in the DNBC  **e** Four individuals were excluded due to very small missing values **f** Number of parental questionnaires returned and completed, 98.3% filled out by the biological mother, 1.2% by the biological father, 0.1% by others (none biological father or mother, grandparents)  and 0.4 unknown  **g** Includes 3383 individuals excluded due to returning incomplete questionnaires

The DNBC consists of approximately 100,000 pregnancies enrolled via general practitioners from across Denmark in 1996–2003 [17]. Mothers and their offspring have participated in multiple follow-ups, with the most recent 18 years after offspring birth. At the 11-year follow-up (2010–2014), approximately half of the caregivers (mostly mothers) responded to an online questionnaire regarding their child’s health and a wide range of questions regarding housing and indoor home environment at the time their child turned 11 years. At the 18-year follow-up, DNBC children were invited to reply to an online questionnaire in which questions on asthma diagnosis, medication and symptoms were asked. Individuals who had relevant data available from the 11-year and 18-year follow-ups were included in this study (Fig. 1).

#### Indicator variables

Parentally reported data on offspring’s housing conditions in the 11-year follow-up of the DNBC were included as indicators in the LCA model. About forty questions related to housing and indoor home environment were included in the 11-year follow-up questionnaire [18]. This study focuses on the following items:

#### Indoor characteristics

Second hand smoking (SHS) anywhere inside the house *(yes/no)*, winter candle-burning *(seldom/often)*, exhaust hood use *(never-rarely/often-always)*, type of cooking stove *(electrical/gas)*, fireplace use *(no-rarely/yes: weekly or daily)*, mold in child’s bedroom *(yes/no)*, mold in other rooms (yes/no), moisture in child’s bedroom *(yes/no)*, moisture in other rooms (yes/no), flooding *(yes/no)*, cats and dogs ownership *(yes/no),* ownership of other pets (yes/no)*.*

#### Housing characteristics

Housing age *(old* < *1994/new* ≥ *1994)*,^1^ housing type *(apartment/house (detached and semi-detached)/farm)*, ownership *(own/rent)*, household density gathered from two variables, dwelling size and number of individuals in the household. The size of the dwelling was reported as square meters, in intervals of 10, so the middle value was taken for all intervals between the lowest and highest value intervals. Household density (individuals/square meter) was divided into tertiles and then dichotomized *(low-medium/high)*.

#### Outcome

Following the definition by the MeDALL consortium [19], self-reported current asthma at 18-year was defined based on answering yes to any two of the following three questions: “Have you had wheezing or whistling breathing in the past 12 months”; “Has a doctor ever told you that you had asthma?”; “Are you currently taking medicine for your asthma (inhalators, spray or pills) (Supplementary Table 1).

#### Covariates

The following covariates were selected *a priori*: Offspring’s sex was dichotomized as male or female based on data from the Danish Civil Registration System (DCRS).

Information about child’s age when moving to the current dwelling were retrieved from the DNBC 11-year questionnaire “F025 How old was [child name] when you moved to your current home?”. The variable was dichotomized as (*moved/stayed*). Individuals who moved after their first birthday were excluded to ensure residential indoor characteristics did not vary substantially during childhood.

Data on the highest attained maternal education, the year before offspring’s birth in the DNBC, were obtained from the Population Education’s Register. Educational level was classified according to the International Standard Classification of Education (ISCED) version 2011, as low (ISCED 0–2), medium (ISCED 3–4), and high (ISCED 5–8) [18]. The highest attained maternal education the year before offspring’s birth was chosen, as the majority of women’s highest education achievement did not change between offspring’s birth and the child’s 11th birthday.

Data on equivalized total disposable household income for the year before offspring's  birth in the DNBC were obtained from the Income Statistics Register [20]. Quartiles were created for each year of study enrolment. The year before birth was chosen since individuals who moved during childhood (after age one) were excluded. This means that purchasing power in the form of equivalized household income around time of birth will generally be most relevant. Additionally, income is more stable prior to child birth than shortly thereafter, due to temporary decreases in income during parental leave [18].

Several maternal and paternal non-communicable diseases (asthma, diabetes, mental disorders, allergies and cardiovascular disease (CVD)), which have been reported to be determinants of housing choices and behaviors influencing indoor environment [18], were retrieved from the 11-year follow-up questionnaire and, for mothers only, combined with diagnoses from the Danish National Patient Register (DNPR) (Supplementary Table 2) [21].

From the Danish medical birth register [22] we retrieved the following variables: Maternal smoking during pregnancy defined as maternal active smoking during early pregnancy (yes/no); parity (nulliparous/parous); Maternal age at delivery (≤ 25/26–30/31–35, > 35); gestational age at birth (*term/preterm* < 37 weeks of gestation); year of birth (1996–2003) and season of birth defined as follows: *Winter* (December, January, February), *Spring* (March, April, May), *Summer* (June, July, August), *Fall* (September, October, November).

Asthma among the child’s siblings was retrieved from the DNBC 11-year questionnaire “F098 How many of [child name]’s full siblings (biological) have ever had asthma?” and was dichotomized as *(yes/no)* answer.

Offspring smoking status at 18 years was retrieved from the DNBC 18-year questionnaire and was dichotomized as *(yes/no).*

### Statistics

#### Inverse probability weighting

Because participants in the DNBC constitute a selected sample of the general population [23], we performed a loss to follow-up analysis exploring the extent to which our study population, at the 11-year and 18-years follow-up, differed on several important characteristics from those lost to follow-up. In addition, we used inverse probability weighting (IPW) [24] using a reference population, referred to as the eligible population, consisting of all children born in Denmark between 1 June 1997 and 2003, alive and residing in Denmark at their 18th birthday (n = 438,697) (Supplementary Fig. 1). The probability of participation in the study was estimated for each individual using a given set of variables (offspring sex, gestational age at birth, parity, maternal education at birth, maternal age at delivery, maternal smoking and equivalized household income the year before birth) predicting selection into the cohort and loss to follow-up. These variables were obtained from Statistics Denmark and therefore available for all participants as well as non-participants. We estimated a weight for each child (i.e., the inverse of the probability of selection) such that each participant represented not only themselves but also children with similar characteristics that did not participate in the study. Further, we estimated the weights based on the best possible set of existing prediction variables for individuals for whom some of the prediction variables were missing.

#### Latent class analysis

**Fig. 2:**
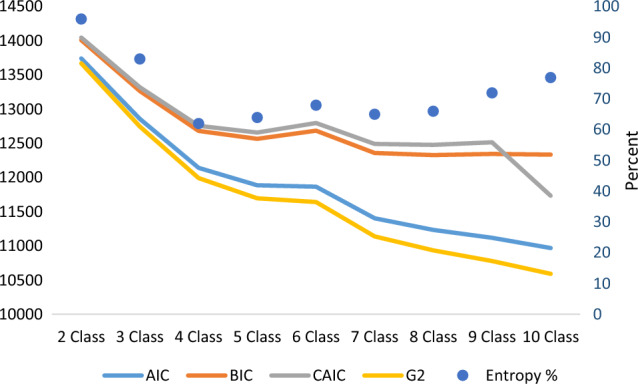
Elbow plot of information criteria values for all latent class analysis models

To address the first aim of the study, to identify latent classes (i.e., clusters) of children from the DNBC who share similar patterns of exposure to indoor pollutant sources, we performed the following analyses:

First, we determined the optimal number of latent classes by fitting and comparing models using only the indicator variables (*secondhand smoking indoor, winter candle-burning, exhaust hood use, type of cooking stove, fireplace use, mold in child’s bedroom, mold in other rooms, moisture in child’ bedroom, moisture in other rooms, flooding, cats and dogs’ ownership, ownership of other pets, housing age, housing type, ownership, household density*) without covariates included in the model (Fig. 2). The following criteria for comparing latent class solutions were used: (a) multiple fit statistics starting with 2 classes up to 10 classes based on goodness-of-fit statistics such as G^2^, the Bayesian information criterion (BIC), the Akaike information criteria (AIC) and the Consistant Akaike information criteria (CAIC), and entropy to evaluate the appropriate number of classes as well as (b) theoretical interpretability [14, 15, 25, 26].

To address the second aim of the study, to examine the association between membership in the latent classes and current asthma at 18-year, we used the corrected three-step approach by Bolck, Croon, and Hagenaars (2004) (BCH), as adapted by Vermunt (2010) [27–29]. Using the BCH method, (1) the parameters of the chosen LCA model were first estimated with covariates (*offspring sex, year of birth, season of birth, gestational age at birth, maternal education, maternal smoking, maternal age at delivery, parity, household income, maternal and paternal chronic diseases, asthmatic siblings*), then (2) the posterior probabilities of class membership based on this model were used to compute a special weighting variable using the BCH-Adjusted Modal Assignment. In short, the BCH-Adjusted Modal Assignment calculates and applies weights that correct for misclassification or measurement error [27]. Finally, (3) the expected probability of the distal outcome, current asthma at 18-year, within each latent class was estimated by taking a weighted average of the observed values for all class participants using pairwise comparisons of latent classes with Wald Chi-Squared Test providing odds-ratios (OR) [27].

#### Sensitivity analyses

To assess asthma incidence at the 18-year follow-up, analyses excluding individuals with a diagnosis of asthma at the 11-year follow-up were performed (see Supplementary Table 1 for asthma definition at 11-year follow-up). In addition, another set of analyses assessing current asthma at age 18, including smoking status at age 18 in addition to the other covariates, was conducted.

All analyses were conducted in Stata version 17. The following two plugins were used: Lanza, S. T., Dziak, J. J., Huang, L., Wagner, A. T., & Collins, L. M. (2018). LCA Stata plugin users' guide (Version 1.2.1). University Park: The Methodology Center, Penn State [26]; and, Huang, L., Dziak, J. J., Bray, B. C., & Wagner, A. T. (2017). LCA_Distal_BCH Stata function users’ guide (Version 1.1). University Park, PA: The Methodology Center, Penn State [27]. Available from methodology.psu.edu.

## Results

### Selection of study participants

Among the 90,811 individuals invited to the DNBC 11-year and 18-year follow-ups (the original birth cohort), only 11% were included in this study (N = 10,329, the study population) as illustrated in Supplementary Table 3. The included individuals were more likely to be females, with a mother of higher socioeconomic status (educational level, income) and to be born at term from a parous mother who did not smoke during pregnancy (Supplementary table 3). Hence, all subsequent analyses were weighted to account for selection.

### Description of study population

Among the 10,329 individuals who were included in this study, the majority lived in owned houses built before 1994, with about half reporting owning a cat and/or a dog at age 11 (Table 1). Interestingly, most individuals were not exposed to SHS (84%), cooking fumes (94%) or a gas cooker (91%), but 81% were exposed to winter candle burning and a third were exposed to fireplace use at home. The presence of moisture in the child’s bedroom was relatively frequent (46%), whereas the presence of mold was very rare (1%). Individuals with current asthma at 18 years (7%) were more likely to be female, living in a dwelling without cats and dogs and from a family with a history of asthma (maternal, paternal and siblings) and allergies (maternal and paternal). The prevalence of other parental diseases did not significantly differ between asthmatic and non-asthmatic children (Table 1).

**Table 1:** Baseline characteristics of the study population for all and by offspring asthma status at age 18

Characteristics	N (%)	Active asthma at age 18		
		Yes (%)	No (%)	*p*-value^a^
Total	10,329 (100)	738 (7)	9591 (93)	
*Offspring characteristics at birth*				
Sex				
Male	4347 (42)	264 (36)	4083 (43)	
Female	5982 (58)	474 (64)	5508 (57)	< .001
Year of birth				
1996 -1997	115 (1)	9 (1)	106 (1)	
1998	1224 (12)	102 (14)	1122 (12)	
1999	2682 (21)	168 (23)	2214 (23)	
2000	2482 (24)	170 (23)	2282 (24)	
2001	2126 (21)	149 (20)	1977 (21)	
2002	1726 (17)	121 (16)	1605 (17)	
2003	304 (3)	19 (3)	285 (3)	.85
Season of birth				
Winter	2349 (23)	174 (24)	2175 (23)	
Spring	2702 (26)	199 (27)	2503 (26)	
Summer	2746 (27)	173 (26)	2554 (27)	
Fall	2532 (25)	173 (23)	2359 (25)	.82
Gestational age at birth				
Term	9940 (96)	703 (95)	9237 (96)	
Preterm (< 37 completed weeks)	389 (4)	35 (5)	354 (4)	.15
*Offsprings’ residential indoor characteristics at 11-year follow-up*				
Indoor *SHS*				
Yes	581 (6)	37 (5)	544 (6)	
No	9748 (94)	701 (95)	9047 (94)	.45
Exhaust hood use				
Often/always	9751 (94)	699 (95)	9052 (94)	
Never/rarely	578 (6)	39 (5)	539 (6)	.70
Type of cooking stove				
Electric	9401 (91)	668 (91)	8733 (91)	
Gas	928 (9)	70 (9)	858 (9)	.62
Fireplace use				
Yes	3381 (33)	228 (31)	3153 (33)	
No	6948 (67)	510 (69)	6438 (67)	.27
Winter candle-burning				
Seldom	1959 (19)	145 (20)	1814 (19)	
Often	8370 (81)	593 (80)	7777 (81)	.62
Ownership				
Rent	652 (6)	51 (7)	601 (6)	
Own	9677 (94)	687 (93)	8990 (94)	.49
Housing type				
Apartment	443 (4)	32 (4)	411 (4)	
House	8178 (79)	608 (82)	7570 (79)	
Farm	1708 (17)	98 (13)	1610 (17)	.05
Building year				
< 1994	9031 (87)	655 (89)	8376 (87)	
≥ 1994	1298 (13)	83 (11)	1215 (13)	.62
Household density				
Low/medium	7888 (76)	565 (77)	7323 (76)	
High	2441 (24)	173 (23)	2268 (24)	.89
Flooding				
Yes	1828 (18)	145 (20)	1683 (18)	
No	8501 (82)	593 (80)	7908 (82)	.15
Moisture in child’s bedroom				
Yes	4753 (46)	338 (46)	4415 (46)	
No	5576 (54)	400 (54)	5176 (54)	.90
Moist in other rooms				
Yes	890 (9)	51 (7)	839 (9)	
No	9439 (91)	687 (93)	8752 (91)	.09
Mold in child’s bedroom				
Yes	97 (1)	11 (1)	86 (1)	
No	10,232 (99)	727 (99)	9505 (99)	.11
Mold in other rooms				
Yes	606 (6)	43 (6)	563 (6)	
No	9723 (94)	695 (94)	9028 (94)	.96
Cats or dogs (indoors)				
Yes	5319 (52)	317 (43)	5002 (52)	
No	5010 (49)	421 (57)	4586 (48)	< .001
Other pets (indoors)				
Yes	1689 (16)	127 (17)	1562 (16)	
No	8640 (84)	611 (83)	8029 (84)	.51
*Offspring characteristics at 18-year follow-up*				
Smoking status at 18-year				
Yes	1901 (18)	166 (23)	1735 (18)	
No	8428 (82)	572 (77)	7856 (82)	.003
*Maternal characteristics before or at time of offspring’s birth*				
Maternal education level^b^				
Low	628 (6)	46 (6)	582 (6)	
Medium	4382 (42)	309 (42)	4073 (43)	
High	5319 (52)	383 (52)	9591 (52)	.95
Maternal smoking during pregnancy				
No	9311 (90)	655 (89)	8656 (90)	
Yes	1018 (10)	83 (11)	935 (10)	.19
Maternal age at delivery (years)				
≤ 25	408 (4)	32 (4)	376 (4)	
26–30	3603 (35)	268 (36)	3335 (35)	
31–35	4421 (43)	324 (44)	4097 (43)	
> 35	1897 (18)	114 (15)	1783 (19)	.20
Parity				
Nulliparous	3160 (31)	228 (31)	2932 (31)	
Parous	7169 (69)	510 (69)	6659 (61)	.85
Equivalized household income at birth^c^				
1st quartile (lowest)	795 (8)	55 (8)	740 (8)	
2st quartile	2191 (21)	171 (23)	2020 (21)	
3st quartile	331 (32)	243 (33)	3068 (32)	
4st quartile (highest)	4032 (39)	269 (36)	3763 (39)	.38
*Familial non-communicable diseases status at 11-year follow-up*				
Maternal NCDs				
Asthma				
Yes	885 (9)	114 (15)	771 (8)	
No	9444 (91)	624 (85)	8820 (92)	< .001
Diabetes				
Yes	177 (2)	12 (2)	165 (2)	
No	10,152 (98)	726 (98)	9426 (98)	.85
Mental disorders				
Yes	1160 (11)	97 (13)	1063 (11)	
No	9169 (89)	641 (87)	8528 (89)	.08
Allergies				
Yes	3445 (33)	302 (41)	3143 (33)	
No	6884 (67)	436 (59)	6448 (67)	< .001
CVD				
Yes	1277 (12)	107 (14)	1170 (12)	
No	9052 (88)	631 (86)	8421 (88)	.07
Paternal NCDs				
Asthma				
Yes	1278 (12)	186 (25)	1092 (11)	
No	9051 (88)	552 (75)	8499 (89)	< .001
Diabetes				
Yes	539 (5)	39 (5)	500 (5)	
No	9790 (95)	699 (95)	9091 (95)	.93
Mental disorders				
Yes	867 (8)	74 (10)	793 (8)	
No	9462 (92)	664 (90)	8798 (92)	.10
Allergies				
Yes	334 (32)	337 (46)	2997 (31)	
No	6995 (68)	401 (54)	6594 (69)	< .001
CVD				
Yes	1976 (19)	151 (20)	1825 (19)	
No	8353 (81)	587 (80)	7766 (81)	.34
Asthma among siblings				
Yes	387 (4)	119 (16)	268 (3)	
No	9942 (96)	619 (84)	9323 (97)	< .001

*NCD* non-communicable diseases; *CVD* cardiovascular diseases; *SHS* second hand smoke

^a^chi-squared tests

^b^^−^^c^Measured the year before offspring’s birth

### Choosing the number of latent classes in LCA

To determine the number of latent classes within our dataset, we compared goodness-of-fit measures for models with 2–10 latent classes (Fig. 2). We selected our final model with five latent classes because that choice produced the lowest BIC and CAIC followed by an increase in the model with six classes, as shown in the elbow plot in Fig. 2. The five-class model was also found to be the best in terms of theoretical interpretability. The entropy of the five class model was 0.64, which indicates that the classification accuracy of the five classes model is moderate [25].

### Residential indoor characteristics by class membership

**Fig. 3:**
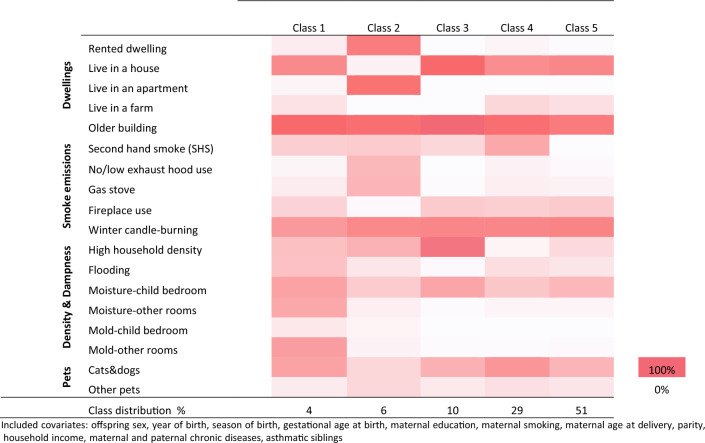
Heat map of the distribution of residential indoor characteristics (item response probabilities) by latent class of 10,329 children participating in the 11-year and 18-year follow-ups of the DNBC

The distributions of residential indoor characteristics by classes, which are referred to as item-response probabilities, are shown in Fig. 3 and in supplementary table 4 for the LCA model with covariates. Covariates included in the model were as follows:  offspring sex, year of birth, season of birth, gestational age at birth, maternal education, maternal smoking, maternal age at delivery, parity, household income, maternal and paternal diseases and asthmatic siblings.

Across classes, the distribution of item-response probabilities was homogenous for winter candle burning, living in a dwelling built before 1994, mold in child’s bedroom and living with other pets, respectively.

The following patterns of clustering for each class were observed. The first class, including 4% of our total sample, was characterized by children living in older owned houses exposed to winter candle burning, higher exposure to flooding, moisture in child's bedroom as well as mold and moisture in other rooms, fireplace use and cats and/or dogs’ ownership. Compared to other classes, clustering on items related to household dampness was strong. The second class, 6% of the sample, was characterized by children living in older rented apartments exposed to winter candle burning, lower exhaust hood use, higher gas stove use, but low fireplace use, and higher density. Compared to other classes, clustering on items related to gaseous and particle emissions was strong, especially winter candle burning, lower exhaust hood use, higher gas stove use, although fireplace use was particularly low (only 3%). This could partly be explained by the vast majority of individuals in class two living in rented apartments. Ten percent of the sample was in the third class, which was characterized by children living in older owned houses with high density, candle burning and to a lower extent, moisture in the child’s bedroom and cat and dog ownership. Compared to other classes, the density item was very high. The fourth class, which was the second biggest representing 29% of the sample, also included children living in older owned houses (67%), or in a farmhouse (32%) exposed to winter candle burning and high probability of pet ownership. Compared to other classes, the probabilities of pet ownership (cats and dogs as well as other pets) and living in a farmhouse were higher. The fifth class, representing 51% of the sample, was characterized by children living in older owned houses, exposed to winter candles burning, moisture in child’s bedroom and fireplace use. No clear clustering pattern could be identified in this class.

### Latent class membership and current asthma at 18 years

The first part of Table 2 shows the estimated risk (probability) of current asthma in each latent class in the LCA models both with and without covariates. Here we describe the results of the model with covariates. The data suggest that the probability of current asthma at 18 years was highest among individuals in class one with higher clustering on household dampness (9, 95%CI 0.06–0.13), followed by individuals in class five with no clear clustering pattern (8, 95%CI 0.07-0.09) and lowest in class four with higher clustering on pets’ ownership and living in a farm (5, 95%CI 0.04–0.07).

**Table 2:** Association between latent class membership and current asthma at 18 years of 10,329 children participating in the 11-year and 18-year follow-ups of the DNBC

	Model without covariates			Model with covariates ^*^		
	*Estimated probabilities of asthma in each of the latent class*					
Class	Probability	SE	95%CI	Probability	SE	95%CI
1	.09	.02	.06 -.13	.09	.02	.06 -.13
2	.07	.01	.04—.09	.07	.01	.05—.10
3	.07	.01	.05—.08	.07	.01	.05—.09
4	.05	.01	.03—.06	.05	.02	.04—.07
5	.09	.01	.07—.10	.08	.01	.07—.09
	*Pairwise comparisons of latent classes using odds-ratios (OR)*					
	OR	95%CI	*p*-value	OR	95%CI	*p*-value
Class 2 vs 1	0.71	0.39–1.31	.28	0.76	0.43–1.34	0.34
Class 3 vs 1	0.69	0.40–1.17	.16	0.70	0.40–1.21	0.19
**Class 4 vs 1**	**0.46**	**0.26–0.84**	**.01**	**0.53**	**0.32–0.88**	**0.01**
Class 5 vs 1	0.90	0.55–1.47	.68	0.86	0.56–1.33	0.5
Class 3 vs 2	0.96	0.60–1.54	.88	0.92	0.49–1.72	0.74
Class 4 vs 2	0.65	0.38–1.13	.13	0.70	0.43–1.14	0.15
Class 5 vs 2	1.26	0.76–2.10	.28	1.14	0.75–1.72	0.54
Class 4 vs 3	0.68	0.41–1.10	.12	0.76	0.50–1.17	0.22
Class 5 vs 3	1.31	0.98–1.768	.07	1.24	0.84–1.84	0.27
**Class 5 vs 4**	**1.94**	**1.19–3.17**	**.01**	**1.63**	**1.12–2.36**	**0.01**

The first part of the table shows estimated probabilities of asthma in each of the latent class and the second part pairwise comparisons of latent classes using odds-ratios

^*^Included covariates: offspring sex, year of birth, season of birth, gestational age at birth, maternal education, maternal smoking, maternal age at delivery, parity, household income, maternal and paternal chronic diseases, asthmatic siblings

In the second part of Table 2, the odds of current asthma is compared between the latent classes. Consistently with the first part of the table, individuals in class four (with higher clustering on pets’ ownership and living in a farm) had lower odds of current asthma at age 18 compared to individuals in class one (with higher clustering on household dampness) (OR 0.53 (95%CI 0.32–0.88), *p* = 0.01). On the other hand, individuals in class five had increased odds of current asthma at age 18 compared to individuals in class four (OR 1.63 (95%CI 1.12–2.36) *p* = 0.01).

### Sensitivity analysis

The probability of incident asthma at age 18 was lower than current asthma and homogenous across classes. The associations between latent class membership and asthma incidence from age 11 to age 18 years did not indicate that one class had higher or lower odds compared to another (Supplementary table 5).

Including smoking at age 18 in the model essentially showed similar results to the main analysis (Supplementary table 6).

## Discussion

To add to the conversation on how similar exposure profiles of home indoor characteristics during mid-childhood associated with asthma during adolescence, we applied LCA to identify groups of individuals with similar patterns of home characteristics. We identified five groups of different patterns of residential indoor characteristics.

There were limited differences in the probability of current or incident asthma at age 18 between classes. Nevertheless, the odds of current asthma were highest among individuals belonging to class one which was characterized by higher exposure to dampness at home during childhood. Several previous meta-analyses and epidemiological studies have found a positive association between the presence of moisture and mold in homes, either measured or self-reported, and the risk of childhood asthma or markers thereof [30–37]. Our findings suggest that the presence of moisture and mold in the child’s bedroom might be most important in regard to active offspring asthma at age 18. Similarly, four Finnish studies have also found that moisture damage or visible mold in the child's main living areas, including the child's bedroom, were more strongly associated with asthma prevalence, persistent asthma as well as fractional exhaled nitric oxide, a marker of asthma [38], compared to moisture damage or visible mold in other areas of the house [31–33, 39]. On the other hand, regarding mold damage remediation in houses and decreases in asthma-related symptoms, use of asthma medication in asthma patients and respiratory infections, a Cochrane review from 2015 concluded that there was only moderate-quality evidence and that more randomized studies were needed. The authors also noted that one of the reasons for null findings from remediation studies might be explained by the fact that bronchial asthma is a chronic disease which is not quickly reversible [40]. Several possible mechanisms have been suggested for the associations between home dampness or indoor moisture and mold and asthma. The presence of indoor moisture and mold enables fungal growth. According to a meta-analysis and systematic review on indoor fungal diversity and asthma, the type of fungi that might play a role in the development of asthma are primarily Penicillium, Aspergillus, and Cladosporium [41]. Although a relationship between exposure to microorganisms and IgE sensitization has been reported, mechanisms behind the adverse health effects of moisture damage and mold may not be IgE mediated [42–44]. Indeed, exposure to moisture damage or mold may cause allergy-like symptoms due to histamine release without measured IgE levels. Furthermore, components of fungal cell walls, such as volatile organic compounds, may act as irritants in the airways. Asthma development and exacerbation, might therefore be caused my repeated irritation in the respiratory tract [42–44].

Our results also suggest that living in a farmhouse and owning pets during childhood might be protective factors against current asthma at age 18. Indeed, the lowest probability of current asthma at age 18 was observed in class four where one third of individuals lived in a farmhouse and the majority owned pets. Living in a farmhouse and owning pets clustered together and were associated with lower current asthma at age 18, and this was especially true when compared to the class with the highest proportion of individuals who lived in a dwelling characterized by high dampness (class 1). Multiple worldwide epidemiological studies indicate that children growing up on farms are less susceptible to allergic diseases and asthma [45–47], and a recent study among > 30,000 individuals showed an inverse association of farm upbringing on the risk of asthma [48]. Although it has not yet been possible to elucidate every crucial variable related to the observed ‘farm effect’, the microbial environment of the farm during pregnancy and early childhood has been shown to be protective against atopy [47]. Farm-related exposures have been shown to shape children's immune homeostasis, via mediators such as N-glycolylneuraminic acid or arabinogalactan, or by diverse environmental microbes. Farm-related exposures induce an anti-inflammatory response of the innate immunity and increase the differentiation of regulatory T cells and T helper cell type 1 [49]. Moreover, nutritional factors, such as breastfeeding or farm milk and food diversity, inducing short-chain fatty acids-producing bacteria in the intestine, might contribute to ‘farm effects’ [49]. Another important factor might be the presence or absence of livestock on the farm, as Downs et al. found that exposure to farms with livestock was more likely to convey protection against atopic disease than farms without animals [50]. Regarding pet ownership, our results also show that individuals with current asthma at 18 years were more likely to have been living in a dwelling without cats and dogs. This last finding is consistent with our previous descriptive study on indoor home environments of Danish children and the socioeconomic position and health of their parents which showed that the proportion of pet ownership was lower among households where parental asthma or allergies and atopy were prevalent [17]. Currently, the evidence regarding the association between pet ownership and asthma is unclear. However, a recent large meta-analysis of primary data suggests that cat and dog ownership may exacerbate the risks associated with pet-specific sensitization but offer some protection against asthma in the absence of sensitization [51]. Although we did not distinguish between farms with and without livestock, our results suggest that the combination of being exposed to the ‘farm effect’ and animals (pets) during childhood might be especially beneficial with regards to asthma development.

Individuals belonging to class 2 characterized by higher exposure during childhood to cooking fumes, from gas stove usage and low exhaust hood use were not at increased odds of asthma during adolescence, compared to other classes in our study. These results, although counter-intuitive, might be explained by different factors. Gas stoves used for cooking without fume hoods or ventilation are an important source of ultra-fine particles (UFPs), NO_2_, carbon monoxide (CO), and formaldehyde (HCHO) [52]. A review from 2008 concluded that although a large number of studies have investigated the relationship between gas stoves and asthma prevalence or asthma symptoms, results were inconsistent, especially among adults [53]. One reason might be linked to gas stoves being used for shorter periods of time and with their use being limited to the kitchen. Additionally, in a randomized controlled double-blinded crossover study among 36 young non-smoking Danes with asthma, short-term exposure to emissions from cooking and candles exerted mild inflammation in males with asthma and decreased comfort among males and females with asthma. However, no changes were observed in lung function measurements (FEV1 and FVC) comparing cooking and candle exposures to clean air [54]. These results suggest that lung function measurements may be less sensitive to short-term air-pollution exposures. The relationship between cooking fumes and asthma might also be context-specific. Factors such as smaller dwelling size, density, and inadequate exhaust hood use contribute to elevated concentrations of NO_2_ in lower-income, multifamily buildings [55]. In addition, according to a review from 2020 on the adverse health effects associated with household air pollution from cooking or heating facilities, the burden of disease was almost exclusively borne by low and middle-income countries [56]. Although individuals in class 2 were potentially more exposed to cooking fumes, the usage of fireplace was the lowest in this class compared to the four others (3% vs. 26–35%), suggesting lower exposure to pollutants emitted by fireplaces. These factors might contribute to explaining our findings.

Indoor candle burning, which is a source of indoor ultrafine particles and NO_2_, is very popular in Denmark. A previous study found that candle burning was responsible for more than half of the residential daily integrated exposure to particle concentration in Danish homes where candle burning took place [57]. In our study, individuals among all classes were exposed to candle burning related pollution. Hence, the homogeneity of item-response probability for candle burning across classes might have attenuated the true association between sources of smoke emissions, which emits ultrafine particles, and asthma in adolescence. On the other hand, the use of candles in wintertime in a cohort of middle-aged men and women was not associated with lower lung function or risk of developing asthma [58, 59].

### Strengths and limitations

Our cohort of more than 10,000 children, with data on a large number of indoor home characteristics collected at around age 11, linkage to administrative registry data as well as prospectively collected data on asthma, is a strength. Nevertheless, other meaningful data on indoor characteristics such as ventilation and heating installations, building materials, use of cleaning products, seasonal variations in indoor activities, renovations and refurbishing as well as specific pollutants and time-activity patterns were not available. Although data collection on indoor home variables at 11 years most likely reflect exposure throughout childhood, earlier and repeated data collection would have been better as asthma onset earlier in childhood is common. Additionally, the interactions between outdoor and indoor environment as well as dietary variables were not examined here. Due to its design, causality may not be inferred from this study. However, due to its prospective nature and the inclusion of parental chronic diseases in the model, which have been shown to influence indoor and housing characteristics, reverse causation is unlikely.

In this study we chose to use parent-reported and self-reported data on asthma from the 11-year and 18-year follow-ups, respectively. The use of self-reported data over register-based data was constrained by data access restriction. A study comparing three different asthma classification methods, namely parental-report of doctor-diagnosed asthma of the child from the DNBC 7-year follow-up, hospitalization registry, and prescription registry, yielded low agreement between the three measures and a substantial non-overlap between cases identified which reflect that these definitions represent three distinct phenotypes [60].

Attrition in the cohort was associated with poorer socioeconomic position, male gender and being born preterm from a primiparous mother. However, sample selection was accounted for by using inverse probability weighting [61], as DNBC participants are a selected sample of the source population [23] and many participants did not participate in both the 11-year and 18-year follow-up or had moved during childhood.

By using LCA, a finite set of latent classes characterized by the intersection of numerous characteristics was identified in the present analysis. This approach enabled us to assess the effect of combinations (clusters) of many indicators as opposed to more classical methods where each indicator is singled out. This LCA perspective can provide important information about which residential indoor pollutant sources should be primarily reduced or avoided at home. It may also help in developing targeted interventions, which are expected to show the strongest response, for asthma prevention. One of the weaknesses identified with the use of LCA is that the identified classes may not necessarily refer to existing subgroups within the population [62]. However, to limit misidentification, we used multiple fit statistics, entropy as well as theoretical interpretability when defining the number of classes. In addition, when examining the association between membership in the latent classes and current asthma at 18-year, we corrected for measurement error by using the corrected three-step approach by Bolck, Croon, and Hagenaars (2004) (BCH), as adapted by Vermunt (2010) [27–29].

## Conclusion

Our findings suggest that, in a high-income country such as Denmark, groups of adolescents growing up in homes with mold and moisture during mid-childhood might be at increased odds of current asthma at age 18. On the other hand, teenagers who grew-up in a farm and who were exposed to pets seem less likely to suffer from asthma by age 18. What this study also shows, is that even in the presence of other possible exposures within clusters there are a few key exposures that stand out, which suggests these are more important risk factors to intervene on. These results might be useful for public health interventions aimed at preventing asthma development among children and adolescents.

### Supplementary Information

Below is the link to the electronic supplementary material.

Supplementary file1 (DOCX 58 KB)

## Footnotes

1 The age of the dwelling was reported in intervals (e.g. 1941–1960). We aggregated these into two categories: before 1994, and 1994 and after. The cut-off was chosen based on intervals reported that approximately coincided with the full phasing out of asbestos in construction in Denmark [63]
