# Supplementary material for: Exposure to different residential indoor characteristics during childhood and asthma in adolescence: a latent class analysis of the Danish National Birth Cohort
Source: Eur J Epidemiol. 2023 Oct 21;39(1):51–65. doi: 10.1007/s10654-023-01051-y (PMC10811114; doi:10.1007/s10654-023-01051-y)
Supplement: Supplementary file 1 — Supplementary file1 (DOCX 58 KB) [file 10654_2023_1051_MOESM1_ESM.docx]

**Supplementary documents**

Contents

[Supplementary table 1: asthma case definition 2](#_Toc121483160)

[Supplementary table 2: Parental non-communicable diseases 3](#_Toc121483161)

[Supplementary table 3: Characteristics of the 90’811 individuals invited to participate in the DNBC 11-year and 18-year follow-ups and study population according to follow-up status at age 11 and 18 4](#_Toc121483162)

[Supplementary Figure 1: Flowchart of children born in Denmark between 1^st^ June 1997 and 23^rd^ June 2003 eligible for inclusion in the DNBC 5](#_Toc121483163)

[Supplementary table 4: Distribution of residential indoor characteristics (item response probabilities) by latent class of 10’329 children participating in the 11-year and 18-year follow-ups of the DNBC 6](#_Toc121483164)

[Supplementary table 5: Association between latent class membership and asthma incidence at 18 years of 10’329 children participating in the 11-year and 18-year follow-ups of the DNBC. The first part of the table shows estimated probabilities of asthma in each of the latent class and the second part pairwise comparisons of latent classes using odds-ratios 7](#_Toc121483165)

[Supplementary table 6: Association between latent class membership and current asthma at 18 years of 10’329 children participating in the 11-year and 18-year follow-ups of the DNBC (including smoking status at age 18). The first part of the table shows estimated probabilities of asthma in each of the latent class and the second part pairwise comparisons of latent classes using odds-ratios 8](#_Toc121483166)

# Supplementary table 1: asthma case definition

|  | DNBC 18-year follow-up questionnaire |
| --- | --- |
|  | Have you had wheezing or whistling breathing within the past 12 months? |
|  | Has a doctor ever told you that you had asthma? |
|  | Are you currently taking medicine for your asthma (inhalators, spray or pills)? |
|  | Asthma definition at 18-year: answering yes to any two of the above questions |
|  |  |
|  | DNBC 11-year follow-up questionnaire |
|  | 1.1 Has [child name] ever had wheezing or whistling breathing during the past year? |
|  | 1.2 Has [child name] ever had asthma? |
|  | 1.3 Has [child name] been given medicine for [his/hers] wheezy breathing or asthma (e.g. inhalators, spray or pills) in the past year? |
|  | Asthma definition at 11-year: 1.1 and 1.2 or 1.3 |

# Supplementary table 2: Parental non-communicable diseases

| **Disease** | **Maternal** | **Paternal** |
| --- | --- | --- |

|  | ICD-8 codes | ICD-10 codes | Questionnaire | Questionnaire |
| --- | --- | --- | --- | --- |
| Asthma | 493 | J45 | F202_7 Asthma |  |
| Diabetes | 250 | E10-E14 | F202_1 T1D  F202_2 T2D | F178_1 T1D  F178_2 T2D |
| Mental disorders | 296 | F30-F39; F40-F48 | F202_16 Depression  F202_17 Schizophrenia  F202_18 Other mental disorder | F178_9 Depression  F178_10 Schizophrenia |
| Allergies | 691 | L20 | F202_8 Hay fever  F202_9 Atopic eczema  F202_10 Food allergy  F202_11other allergy | F178_7 Allergy |
| CVD |  | I25.10; I48.91; I63.9; I67.9 | F202_5  High blood pressure  F202_6  High cholesterol | F178_3 Heart attack  F178_4 Stroke  F178_5 High blood pressure |

Questionnaire: 11-year questionnaire; T1D: type 1 diabetes; T2D: type 2 diabetes; CVD: cardiovascular diseases

# Supplementary table 3: Characteristics of the 90,811 individuals invited to participate in the DNBC 11-year and 18-year follow-ups and study population according to follow-up status at age 11 and 18

| **Characteristics** | **Study population (%)** | **Lost to follow-up^a^ (%)** | **p-value** |
| --- | --- | --- | --- |
| Total | 10329 | 80482 |  |
| Offspring sex |  |  |  |
| Male | 4347 (42) | 42182 (52) |  |
| Female | 5982 (58) | 38300 (48) | <.001 |
| Gestational age at birth |  |  |  |
| Term | 9940 (96) | 76811 (95) |  |
| Preterm | 389 (4) | 3671 (5) | <.001 |
| Maternal education level^b^ | | | |
| Low | 628 (6) | 11611 (14) |  |
| Medium | 4382 (42) | 38744 (48) |  |
| High | 5319 (52) | 29817 (37) |  |
| Missing | 0 | 310 (0.4) | <.001 |
| Maternal age at delivery | | | |
| ≤25 | 408 (4) | 10607 (13) |  |
| 26-30 | 3603 (35) | 33321 (41) |  |
| 31-35 | 1121 (43) | 27154 (34) |  |
| >35 | 1897 (18) | 9400 (12) | <.001 |
| Maternal smoking during pregnancy | | | |
| No | 9311 (90) | 64965 (81) |  |
| Yes | 1018 (10) | 14321 (18) |  |
| Missing | 0 | 1196 (1) | <.001 |
| Parity |  |  |  |
| Nulliparous | 3160 (31) | 28789 (36) |  |
| Parous | 7169 (69) | 51580 (64) |  |
| Missing | 0 | 113 (0.1) | <.001 |
| Equivalized household income at birth^c^ | | | |
| 1^st^ quartile (lowest) | 795 (8) | 14286 (18) |  |
| 2^st^ quartile | 2191 (21) | 19937 (25) |  |
| 3^st^ quartile | 3311 (32) | 22312 (28) |  |
| 4^st^ quartile (highest) | 4032 (39) | 23344 (29) |  |
|  | 0 | 603 (0.7) | <.001 |

a We used chi-squared tests of heterogeneity to compare study participants with individuals lost to follow-up.

b-c Measured the year before offspring’s birth

# Supplementary Figure 1: Flowchart of children born in Denmark between 1^st^ June 1997 and 23^rd^ June 2003 eligible for inclusion in the DNBC

443,538 unique mothers

Mothers and children (singletons) alive and living in Denmark at the child’s 18^th^ birthday

or by 31^st^ december of 2015^c^

Children born in Denmark between 1 June 1997 – 23 June 2003 ^a-b^

^a^ The very earliest recruitment to the DNBC was initiated in 1996 as a pilot project in one county. Due to the design and implementation of the pilot project, we were unable to define the eligible population for this period, and this explains the slightly different recruitment period than otherwise reported for DNBC.

^b^ The recruitment to the DNBC was gradually implemented in the 15 counties of Denmark from 1997-1999. The recruitment was nationwide by October 1999 and the eligible population was defined as children born in each county 5 months subsequent to the first date of recruitment and until the date of birth of the last child recruited into the DNBC, which was 23 June 2003.

^c^ The latest available update from registers was in 2015 when most DNBC children had not yet turned 18 years old

452,125 live born children

**Children**:

438,697

**Mothers**:

322,651

**Eligible population**

# Supplementary table 4: Distribution of residential indoor characteristics (item response probabilities) by latent class of 10,329 children participating in the 11-year and 18-year follow-ups of the DNBC

|  |  |  |  |  |  |  |
| --- | --- | --- | --- | --- | --- | --- |
|  |  | Class 1 | Class 2 | Class 3 | Class 4 | Class 5 |
| **Dwellings** | Rented dwelling | 9 | 86 | 9 | 3 | 1 |
|  | Live in a house | 75 | 7 | 85 | 67 | 89 |
|  | Live in an apartment | 4 | 93 | 0 | 0 | 0 |
|  | Live in a farm | 21 | 1 | 16 | 32 | 10 |
|  | Older building | 97 | 96 | 92 | 90 | 83 |
| **Smoke emissions** | Second hand smoke (SHS) | 7 | 17 | 8 | 18 | 0 |
|  | No/low exhaust hood use | 5 | 46 | 5 | 6 | 3 |
|  | Gas stove | 11 | 48 | 5 | 10 | 6 |
|  | Fireplace use | 30 | 3 | 26 | 33 | 35 |
|  | Winter candle-burning | 68 | 78 | 83 | 82 | 80 |
| **Density & Dampness** | High household density | 33 | 50 | 99 | 4 | 15 |
|  | Flooding | 37 | 15 | 15 | 16 | 17 |
|  | Moisture-child bedroom | 59 | 32 | 51 | 37 | 50 |
|  | Moisture-other rooms | 50 | 11 | 8 | 4 | 6 |
|  | Mold-child bedroom | 12 | 4 | 0 | 0 | 0 |
|  | Mold-other rooms | 63 | 9 | 3 | 1 | 2 |
| **Pets** | Cats&dogs | 64 | 26 | 54 | 74 | 40 |
|  | Other pets | 15 | 24 | 15 | 18 | 16 |
|  | Class distribution % | 4 | 6 | 10 | 29 | 51 |

Included covariates: offspring sex, year of birth, season of birth, gestational age at birth, maternal education, maternal smoking, maternal age at delivery, parity, household income, maternal and paternal chronic diseases, asthmatic siblings

# Supplementary table 5: Association between latent class membership and asthma incidence at 18 years of 10,329 children participating in the 11-year and 18-year follow-ups of the DNBC. The first part of the table shows estimated probabilities of asthma in each of the latent class and the second part pairwise comparisons of latent classes using odds-ratios

| **Estimated probabilities of asthma in each of the latent class** | | | | |
| --- | --- | --- | --- | --- |
| Class | Probability | SE | 95%CI |  |
| 1 | .04 | .01 | .02 -.06 |  |
| 2 | .02 | .01 | .01 - .04 |  |
| 3 | .04 | .02 | .02 - .05 |  |
| 4 | .03 | .01 | .02 - .04 |  |
| 5 | .04 | .004 | .03 - .04 |  |
| **Pairwise comparisons of latent classes using odds-ratios (OR)** | | | | |
|  | OR | 95%CI | | p-value |
| Class 2 vs 1 | 0.63 | 0.24-1.67 | | .33 |
| Class 3 vs 1 | 1.02 | 0.46-2.23 | | .97 |
| Class 4 vs 1 | 0.87 | 0.42-1.79 | | .70 |
| Class 5 vs 1 | 0.96 | 0.50-1.88 | | .91 |
| Class 3 vs 2 | 1.62 | 0.73-3.62 | | .24 |
| Class 4 vs 2 | 1.38 | 0.64-2.96 | | .41 |
| Class 5 vs 2 | 1.54 | 0.76-3.11 | | .23 |
| Class 4 vs 3 | 0.85 | 0.50-1.44 | | .55 |
| Class 5 vs 3 | 0.95 | 0.57-1.58 | | .83 |
| Class 5 vs 4 | 1.11 | 0.70-1.78 | | .65 |

Included covariates: offspring sex, year of birth, season of birth, gestational age at birth, maternal education, maternal smoking, maternal age at delivery, parity, household income, maternal and paternal chronic diseases, asthmatic siblings

# Supplementary table 6: Association between latent class membership and current asthma at 18 years of 10,329 children participating in the 11-year and 18-year follow-ups of the DNBC (including smoking status at age 18). The first part of the table shows estimated probabilities of asthma in each of the latent class and the second part pairwise comparisons of latent classes using odds-ratios

|  | **Model with covariates ^*^** | | | |
| --- | --- | --- | --- | --- |
| **Estimated probabilities of asthma in each of the latent class** | | | | |
| Class | Probability | SE | 95%CI |  |
| 1 | .09 | .02 | .06 -.13 |  |
| 2 | .07 | .01 | .05 - .10 |  |
| 3 | .07 | .001 | .05 - .09 |  |
| 4 | .05 | .001 | .04 - .06 |  |
| 5 | .08 | .001 | .07 - .09 |  |
| **Pairwise comparisons of latent classes using odds-ratios** | | | | |
|  | OR | 95%CI | | p-value |
| Class 2 vs 1 | 0.76 | 0.43-1.34 | | .33 |
| Class 3 vs 1 | 0.72 | 0.42-1.25 | | .24 |
| Class 4 vs 1 | 0.52 | 0.31-0.86 | | .01 |
| Class 5 vs 1 | 0.86 | 0.56-1.32 | | .48 |
| Class 3 vs 2 | 0.96 | 0.58-1.59 | | .87 |
| Class 4 vs 2 | 0.68 | 0.42-1.11 | | .12 |
| Class 5 vs 2 | 1.13 | 0.75-1.71 | | .55 |
| Class 4 vs 3 | 0.71 | 0.46-1.09 | | .12 |
| Class 5 vs 3 | 1.18 | 0.85-1.65 | | .38 |
| Class 5 vs 4 | 1.67 | 1.17-2.37 | | .06 |

Included covariates: offspring sex, year of birth, season of birth, gestational age at birth, maternal education, maternal smoking, maternal age at delivery, parity, household income, maternal and paternal chronic diseases, asthmatic siblings, smoking status at age 18
